# The effect of legal representation on clinical measures in involuntarily admitted psychiatric patients: a retrospective study

**DOI:** 10.1186/s13584-024-00633-9

**Published:** 2024-10-03

**Authors:** Yaacov Cohen, Ariel L. Bendor, Roy Gilbar, Orly Cohen, Razek Khawaled, Arieh Dienstag, Amit Lotan, Omer Bonne

**Affiliations:** 1 Faculty of Law, Bar-Ilan University, Ramat Gan, Israel; 2 School of Law, Netanya Academic College, Netanya, Israel; 3 Department of Biotechnology, Hadassah Academic College, Jerusalem, Israel; 4 Forensic Psychiatry Department, Mental Health Division, Ministry of Health & Hadassah Medical Center, Jerusalem, Israel; 5 Department of Psychiatry, Hadassah Medical Center, Faculty of Medicine, Hebrew University of Jerusalem, Jerusalem, Israel

**Keywords:** Involuntary admission, Revolving door, Legal representation

## Abstract

**Background:**

Most western countries provide funded legal representation (LR) for involuntarily admitted psychiatric patients appearing before judicial committees. In 2004, an amendment to the Israeli Mental Health Act granted this right to involuntarily committed psychiatric patients. Psychiatrists then voiced concerns that LR may increase rates of premature discharge and compromise patients’ safety and well-being. These worries have not been sufficiently addressed to date. This study aimed to provide answers to their concerns.

**Methods:**

This study included 3124 and 3434 inpatients involuntarily admitted to psychiatric facilities in 2000 and in 2010 (respectively), prior to and after the introduction of LR in Israel. Data were acquired from the Israeli National Psychiatric Hospitalization Registry. Clinical measures included percentage of discharges by the District Psychiatric Board (DPB), duration of involuntary hospitalization and rates of readmissions within thirty days and six months of discharge by treating psychiatrists (TP) or DPB.

**Results:**

The odds ratio (OR) of discharge by a DPB in 2010 (n = 221) compared to 2000 (n = 93) was 2.2 [95%CI 1.72–2.82]. The OR was similar for readmissions within thirty days or six months among patients discharged by TP and a DPB (OR = 1.08, *p* = 0.697 and OR = 0.92, *p* = 0.603, respectively) as well as between the two time points (*p* = 0.486 and *p* = 0.618). The duration of hospitalizations terminated by a DPB was significantly shorter than those terminated by TP, with no difference between the study time points. The mean hospitalization duration in 2010 was 21% shorter compared to 2000 among patients discharged by TP.

**Conclusions:**

The number of DPB proceedings and the number of involuntarily hospitalized psychiatric patients discharged by DPBs increased considerably after the advent of state-funded legal representation in 2004. We found that this did not compromise beneficence and non-malfeasance of patient care. Our results emphasize the feasibility of affording even the most severely mentally ill patients the rights to due process. These findings may relieve concerns about state-funded LR procedures in involuntary psychiatric hospitalizations.

**Supplementary Information:**

The online version contains supplementary material available at 10.1186/s13584-024-00633-9.

## Introduction

The legal criteria for involuntary admission to psychiatric facilities in Israel are similar to those of many countries in Europe and the USA [1–4], as well as in Australia and Canada. Testa and West [5] Most European countries require two criteria for the involuntary admission of a patient. The first includes “severe mental disorder”, “psychotic disease” or “need for treatment”, with the purpose of the latter being to prevent real damage due to a mental illness that is debilitating to the extent that it impairs the ability to judge reality [6]. The second criterion is “dangerousness to self or others” [7], with the objective of forced hospitalization being to both enable treatment through the provision of adequate medical care and to protect the public [8]. Involuntary admission based solely upon the need for treatment is exclusive to Italy, Spain, and Sweden, while France, Germany, the Netherlands, USA, Australia [9], Denmark, Finland, Great Britain [10], Greece, Ireland, Portugal and Canada require both criteria, as does Israel [11–13].

In Israel, patients are involuntarily admitted by order of the district psychiatrist in accordance with the Mental Health Act 1991 (MHA) [14], correspondingly with the two criteria mentioned above. The initial commitment order enables coercive hospitalization of up to one week and possible extension by the district psychiatrist for another week if justified by the patient’s clinical condition. The patient has a right to appeal the commitment order to the District Psychiatric Board (DPB), a committee consisting of two psychiatrists and one lawyer who serves as chairperson. Extension of involuntary hospitalization for more than two weeks requires a decision by the DPB, which has the authority to issue a commitment order for up to six months. The DPB plays an essential role in balancing a patient’s right to liberty and in deciding whether the severity of the judgment is proportional under the given circumstances [15]. Although the DPB has a judicial function and can deny a patient’s liberty, Israeli law had not provided patients the right to state-funded legal representation (LR) until 2004.

In 2002, a pilot program was launched by which patients diagnosed with a serious mental illness were granted the option to be represented by a state-funded lawyer during DPB deliberations. In 2004, an amendment to the MHA was passed, granting those patients the right to state-funded LR when appearing before the DPB [14, 16].

The right to LR is derived from the right to due process, which includes a fair hearing [17]. This is especially relevant to involuntarily hospitalized psychiatric patients whose capability to represent themselves appropriately is severely compromised. In order to provide this right to all involuntarily hospitalized psychiatric patients, it is necessary for governments to finance the LR. The role of the representative lawyer is to ensure that a person will be involuntarily hospitalized only if both conditions for coercive hospitalization—mental illness and posing danger to the public—are fully met. If those conditions are not met, the individual may refuse hospitalization even if it is in his/her best medical interests to do so.

Two studies were conducted in Israel at the time of the legislation that gave patients the right to LR [18]. The first compared the rates of readmission within six months among patients discharged by the DPB with LR during the 2002 pilot program to those of 2001 (before LR). The findings showed that readmission rates within six months in patients discharged after being provided LR in the DPB hearings were higher than those for discharged patients who underwent DPB hearings without the benefit of an LR [19]. However, no data on the number of patients discharged were provided, only data on the number of DPB deliberations, and there was no statistical analysis. The second study compared the length of hospitalization and the time from discharge to rehospitalization as well as the percentage of patients that were readmitted within three years of admission between those who had been unrepresented by LR immediately before (n = 108, in 2003–2004) and those represented by LR immediately after (n = 167, in 2005) the initiation of the legislation that gave patients the right to state-funded LR. That study found shortening of the duration of time until readmission in the represented patients if brought before the DPB for appealing involuntary hospitalization (during the first two weeks of admission), and no significant difference (*p* = 0.14) between represented and unrepresented patients if appearing before the DPB during later stages of the hospitalization [20]. Today, nearly all psychiatric patients in Israel are represented by a state-funded lawyer when appearing before the DPB at any stage. Furthermore, the number of DPB hearings has greatly increased since the inception of LR (unpublished and partial data from the files of the Ministry of Health). However, there are no additional studies on the effect of LR upon the length of psychiatric hospitalization in Israel or elsewhere available in the literature. LR is state funded in Israel and in most European countries [18], as well as in Australia and Canada, and there is even a legal obligation for the patient to be represented in some of them [21]. Representation in the USA, however, is funded by the state only in cases where the patient is defined by the court as being “indigent” [3, 22].

**Fig. 1:**
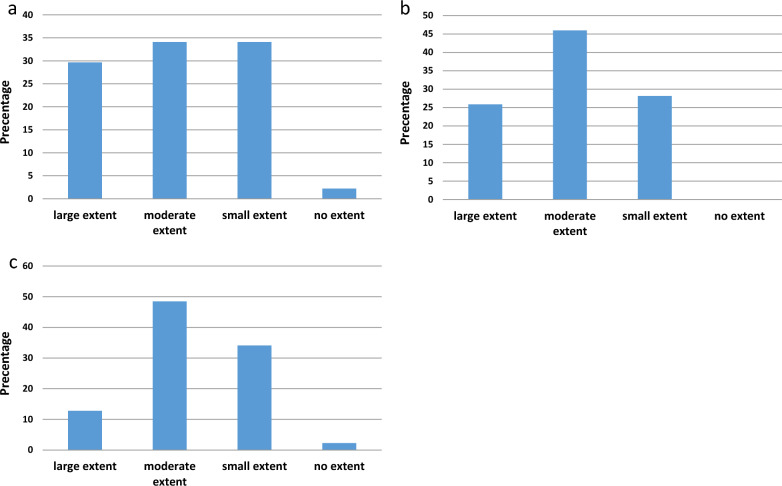
Distribution of responses (in percentage) of practicing Israeli psychiatrists to a survey. The questionnaire was responded to by a sample of 192 psychiatrists representing 52% of the 370 psychiatrists that practice in closed psychiatric wards and/or attend DPBs in Israel. They estimated the degree of influence they believed LR had on **a** increasing earlier termination of psychiatric hospitalization, **b** increasing the RD (**b**), and **c** influencing the DPB’s decisions

We have earlier [23], unpublished) examined the attitudes of practicing Israeli psychiatrists towards LR eleven years after it was introduced. Specifically, we asked them if they believe LR affects the decision of the DPB, causes premature discharge, or increases rates of rapid rehospitalization, often termed the “Revolving Door” phenomenon (RD). A representative sample of 192 psychiatrists (52% of 370 psychiatrists that practice in closed psychiatric wards and/or attend DPBs in Israel) replied to the survey. Around two-thirds (64%) of them responded that the introduction of LR has to a large (30%) or moderate (34%) extent led to the premature termination of psychiatric hospitalization (Fig. 1a). All the respondents believed that LR leads to an increase in RD to some extent (26% to a large extent, 46% to a moderate extent, and 28% to a small extent) (Fig. 1b). Over 60% of the respondents estimated that LR had a large (13%) or moderate (48%) influence on the DPB’s decision, with only 2% responding that LR had no effect at all (Fig. 1c). These findings emphasize the concern among Israeli psychiatrists regarding a potentially deleterious effect of LR. The current study was undertaken to empirically test the abovementioned beliefs. Despite the fact that state-funded LR in psychiatric hospitalizations is now common in most western countries, there is no evidence based data on the effects of LR on rates of discharge and readmission.

## Methods

Data were extracted from the Israeli National Psychiatric Hospitalization Registry of the Ministry of Health, which contains information on all psychiatric hospitalizations in Israel. Psychiatric admissions in 2000 were compared to psychiatric admissions in 2010 (we considered only the first hospitalization for patients who were hospitalized more than once in 2000 or 2010). The year 2000 was selected because it preceded the amendment to the MHA regarding the right to representation by a state-funded lawyer, whereas the year 2010 was selected since LR was an established part of the protocol in all Israeli mental health hospitals by that time point.

Involuntary hospitalizations are terminated for one of the following reasons: first, according to the opinion of treating psychiatrists (TP) that inpatient treatment is no longer clinically warranted; second, according to the opinion of the TP that involuntary inpatient treatment is still warranted but the DPB overruled the decision of the physician; third, according to the opinion of the TP, inpatient treatment is clinically warranted, but the patient insists upon discharge and when conditions for involuntary treatment are no longer met (discharge against medical advice [AMA]); or fourth, emergence of other (often unexpected) circumstances (e.g., medical illness and transfer to a general hospital, died, escaped, etc.). Since we had aimed to estimate the net effect of LR in patients from the second group (PDB-determined discharge) while using the TP’s discharges as a control, the data for patients in the third and the fourth groups did not shed further light on the current study questions, and so patients from the latter two subgroups were excluded from further analyses.

The variables evaluated in this study included duration of involuntary hospitalization, percentage of all discharges by the DPB, and readmissions within thirty days (RD) or six months since discharge. Odds ratios (OR) for the odds of discharge by a TP vs. a DPB in 2010 vs. 2000, or the odds of RD vs. no RD and readmission after six months vs. no readmission after six months among TP- vs. DPB-discharged patients, as well as 95% confidence intervals (CI) were calculated by logistic regression, with frequency weights denoting the number of individuals in each category. The OR for RD and readmission after six months by TP-discharged compared with DPB-discharged patients between 2010 and 2000 were calculated by multiple logistic regression including the year × discharge type interaction term. Log-transformation was applied (Supp. Figure 1b) to assess predictors for duration of hospitalization, given the distributions’ heavy right tails even after censoring at one year (Supp. Figure 1a). Year × discharge type interaction was examined with a two-way ANOVA followed by analysis of simple effects. Data relating to voluntary admissions were similarly derived and used as reference. Statistics were carried out in Stata 16 (StataCorp LLC, USA) and visualized with Prism 9 (GraphPad Software, USA).

## Results

**Fig. 2:**
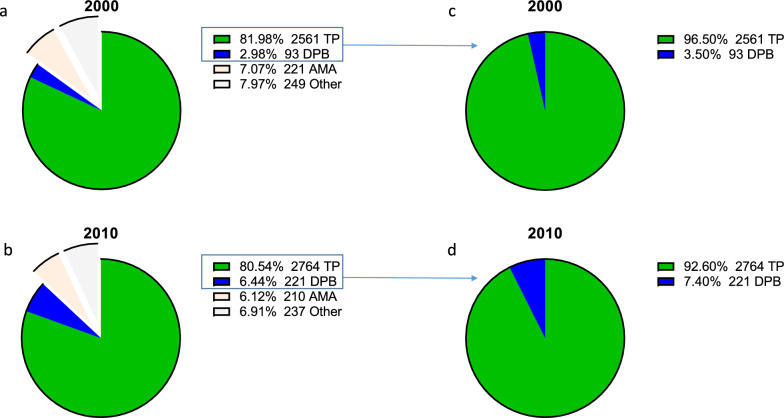
Distribution of patients according to discharge type and year. **a**, **b** Pie diagrams depicting the percentage (number) of patients discharged according to the decision of their treating psychiatrists (TP), by a District Psychiatric Board (DPB), against medical advice (AMA) or due to other reasons during **a** 2000 and **b** 2010. **c**, **d** Similar diagrams following exclusion of patients who were discharged AMA or due to other reasons

In 2000, there were 13,582 psychiatric admissions, of which 3124 were involuntary. In 2010, there were 12,894 psychiatric admissions, of which 3434 were involuntary. Only 5% of the former involuntarily admitted mentally ill patients were represented by LR during their DPB hearing compared to 93.7% in 2010. In 2000, 470 patients (15% of the total annually committed patients) were discharged either AMA or due to emergence of other circumstances, whereas 447 (13% of the total annually committed patients) were discharged for those reasons in 2010 and were excluded from the study (Fig. 2a-b).

**Fig. 3:**
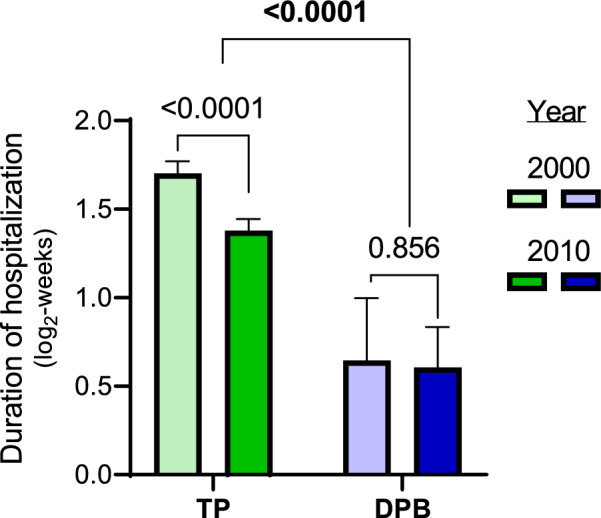
The effects of discharge type and study year on the duration of hospitalization. Bar graph depicting the effects of discharge type (treating psychiatrists [TP] vs. a District Psychiatric Board [DPB]) and of study year (2000 vs. 2010) on mean (± 95% CI) duration of hospitalization (log_2_-weeks). Significance values based upon a two-way ANOVA followed by analysis of simple effects are given

In 2000, 93 (3.5%) of the 2654 civilly committed patients who had been discharged by either TP or a DPB were discharged by a DPB (Fig. 2c). In contrast, 221 of 2985 (7.4%) of those patients were discharged by a DPB in 2010 (Fig. 2d). Accordingly, the OR of discharge by a DPB in 2010 compared to 2000 was 2.2 (95%CI 1.72–2.82) among those that had been involuntarily admitted. The duration of involuntary hospitalizations varied widely during each study year, ranging from several days to several years. Application of log-transformation (Supp. Figure 1b) and a two-way ANOVA model predicting duration of hospitalization by year and discharge type revealed a strong main effect of discharge type upon the duration of hospitalization ((t_5635_ = 8.2, *p* < 0.0001, Fig. 3), with the duration of hospitalizations terminated by a DPB being significantly shorter than those terminated by TP. The year × discharge-type interaction term was not statistically significant (t_5635_ = 1.2, *p* = 0.227). However, examination of simple effects revealed that the mean hospitalization duration among patients discharged by TP in 2010 was reduced by 21% (95%CI 16–26%) compared to 2000 (t_5635_ = − 7.0, *p* < 0.0001). In contrast, the mean hospitalization duration among patients discharged by DPB remained unchanged between the two time points (t_5635_ = − 0.3, *p* = 0.747). As a reference, we note that the mean duration of voluntary hospitalizations in 2010 was 27% (95%CI 24–29%) shorter than in 2000 (t_19,918_ = − 16.3, *p* < 0.0001, Supp. Figure 2).

**Fig. 4:**
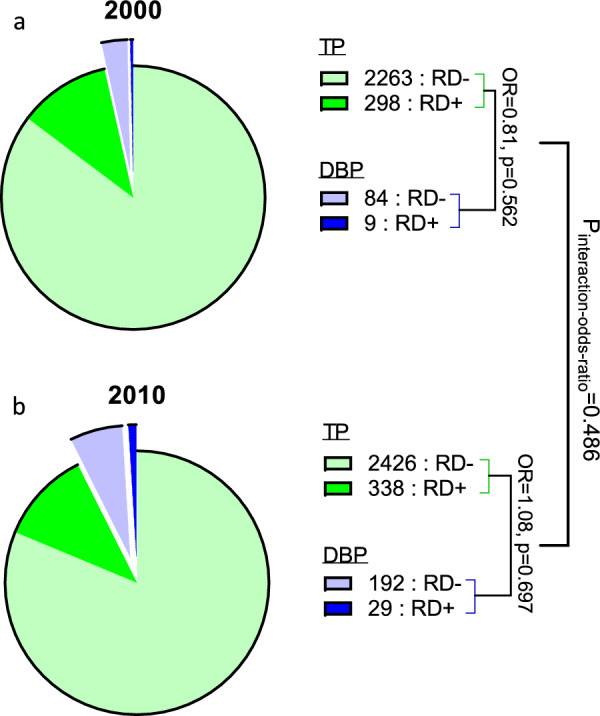
Distribution of patients according to discharge type and study year. Pie diagrams depicting the number of patients displaying RD (i.e., readmission during the immediate 30 days after discharge) according to discharge type (treating psychiatrists [TP] vs. a District Psychiatric Board [DPB]) in **a** 2000 and **b** 2010. The odds ratios (and corresponding significance) for readmission within 30 days among DBP-discharged vs. TP-discharged patients based upon logistic regressions are denoted. The significance of the difference between the odds ratios across the years (P_interaction-ratio_) is based upon a multiple logistic regression model

In 2000, out of 2561 patients discharged by TP following involuntary admission, 298 (11.6%) were readmitted within thirty days of discharge (RD). Of the 93 patients discharged by a DPB during that year, only nine (9.7%) were readmitted within thirty days. The OR for readmission (vs. no readmission) within thirty days among DPB-discharged vs. TP-discharged patients was 0.81 (95%CI 0.41–1.64, Fig. 4a), This means that the odds of early readmission following DPB discharge were slightly (but non-significantly) lower than following TP discharge. In 2010, out of 2764 patients discharged by TP following involuntary admission, 338 (12.2%) were readmitted within thirty days, whereas 29 (13.1%) of the 221 patients discharged by a DPB during that year were readmitted within thirty days. The OR for readmission within thirty days among DPB- discharged vs. TP-discharged patients was 1.08 (95%CI 0.72–1.63, Fig. 4b), meaning that the odds of early readmission following DPB discharge were slightly (although non-significantly) higher than those following TP discharge. Overall, the ratio of these two OR (i.e., interaction “odds ratio”) was non-significant (z = 0.70, *p* = 0.486, Fig. 4), indicating that early readmission rates after discharge from involuntary inpatient admission are similar for discharge by TP and a DPB and remained stable despite the introduction of routine patient legal counselling. For comparison, out of 10,458 patients who had been voluntarily admitted in 2000, 1802 (17.2%) were readmitted within thirty days, while 1366 of the 9462 patients (14.4%) voluntarily admitted in 2010 were readmitted within thirty days. The OR for readmission within thirty days in 2010 compared to 2000 was 0.81 (95%CI 0.75–0.87). Surprisingly, controlling for year, the OR for RD in involuntary vs. voluntary hospitalizations was only 0.72 (95%CI 0.66–0.79).

**Fig. 5:**
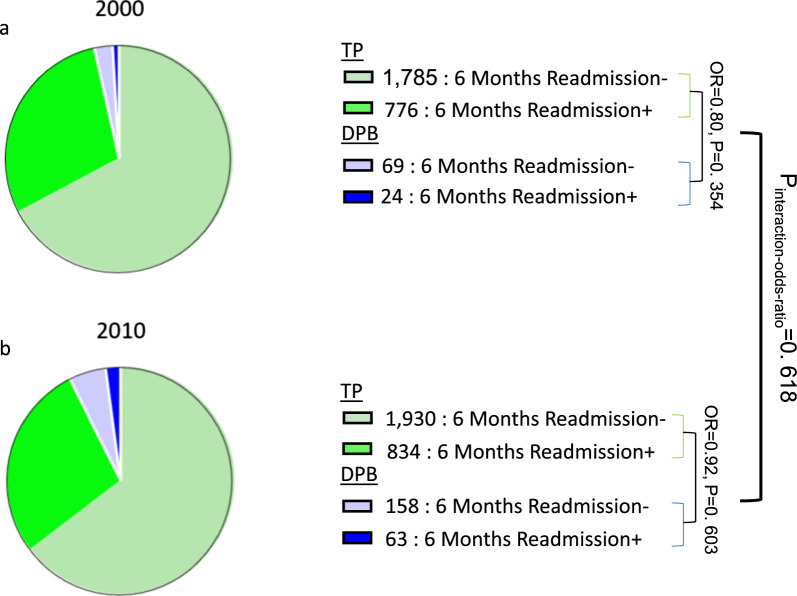
Distribution of patients according to discharge type and study year. Pie diagrams depicting the number of patients readmitted within six months of discharge according to discharge type (treating psychiatrists [TP] vs. a District Psychiatric Board [DPB]) in **a** 2000 and **b** 2010. The odds ratios (and corresponding significance) for readmission within six months among DBP-discharged vs. TP-discharged patients based upon logistic regressions are denoted. The significance of the difference between the odds ratios across the years (P_interaction-ratio_) is based upon a multiple logistic regression model

In 2000, out of 2561 patients discharged by TP following involuntary admission, 776 (30.3%) were readmitted within six months of discharge. Of the 93 patients discharged by a DPB during that year, 24 (25.8%) were readmitted within six months. The OR for readmission (vs. no readmission) within six months among the DPB-discharged vs. the TP-discharged patients was 0.80 (95%CI 0.49–1.28, Fig. 5a), This means that the odds of early readmission following DBP discharge were slightly (but non-significantly) lower than following TP discharge. In 2010, out of 2764 patients discharged by TP following involuntary admission, 834 (30.2%) were readmitted within six months, whereas of the 221 patients discharged by DBP during that year, 63 (28.5%) were readmitted within six months. The OR for readmission within six months for the DPB-discharged vs. the TP-discharged patients was 0.92 (95%CI 0.68–1.25, Fig. 5b), meaning that the odds of early readmission following a DPB discharge were slightly (but non-significantly) lower than following TP discharge. Overall, the ratio of these two OR (i.e., interaction “odds ratio”) was non-significant (*p* = 0.618, Fig. 5), indicating that six-month readmission rates after discharge from involuntary inpatient treatment are similar for discharge by TP and DPBs and remained stable despite the introduction of routine patient LR.

## Discussion

This study was undertaken to address concerns of practicing psychiatrists in Israel regarding putative negative effects of LR in involuntarily hospitalized patients. The legal criteria for involuntary admission as well as the involvement of lawyers in the admission process in Israel is similar to that of most Western countries. Therefore, it is reasonable to assume that concerns raised by Israeli treating psychiatrists as to the intervention of lawyers in their decision-making process (a possible increase in premature discharge and RD) are common to psychiatrists in other Western countries. Our findings show that these concerns are unwarranted. Although more patients were discharged by a DPB in 2010 than in 2000, this had no noticeably negative effect upon the indices examined in the study. Furthermore, the great majority of patients who were presented to a DPB in both years were denied discharge, confirming that involuntary admission meets legal and medical requirements in most cases.

In both 2000 and 2010, the duration of hospitalization in coercively admitted patients was shorter when the decision to discharge was made by a DPB than when it was made by TP. Given that mental health law enables patients to be discharged by TP even if a patient is under a commitment order, the duration of hospitalization upon release by a DPB will inevitably be shorter than the one by TP. Interestingly, while the length of stay in coercively hospitalized patients discharged by TP was considerably shortened in 2010 compared to 2000, no change in length of stay was seen in patients discharged by DPB between these two time points. The mean duration of hospitalization among voluntary admissions was also significantly shorter in 2010 than in 2000, but whether this had any effect on the length of stay of involuntarily hospitalized patients discharged by TP is uncertain.

While the increase in patient release by a DPB in 2010 may be attributable to the introduction of LR, additional factors may also be involved. First, there was a greater number of DPB hearings in 2010. Second, we cannot rule out the possibility that, in 2010, TP may have chosen to bring before a DPB the patients that they consider no longer meet the criteria for involuntary hospitalization in order to transfer the responsibility for discharge to the DPB. We believe this “defensive medicine” approach would more likely have occurred in 2010 rather than in 2000 because of the increase in litigation and “anti-psychiatry” trends propagated by the media. In addition, there may be an improvement in the quality of ambulatory coercive medical care that allows the DPB more flexibility in the practices of discharge from hospitalization in 2010 [24].

Perhaps the most interesting finding of the current study is that neither mode of discharge nor length of hospital stay affected the rates of quick readmission. Worldwide, almost one-seventh of psychiatric patients are readmitted within thirty days of discharge [25]. Our data are similar. Indeed, readmission to a psychiatric hospital within thirty days of discharge is frequently used as an indicator of the quality of hospital care [26–28]. Our data suggest that thirty days readmission as well as six-month readmission rates are unrelated to mode of discharge (TP or DPB) of involuntarily committed patients or even to the nature of the psychiatric hospitalization itself (e.g., by forced commitment or by consent). This may reflect the fact that multiple factors can influence readmission rates, both internal (such as clinical decisions and management protocols) and external (such as societal changes, patient support systems, or legal decisions—in 2010 the psychiatric system may be slower or more hesitant in using its authority to involuntarily hospitalize).

Our data contrast with those of the two studies performed in Israel around the time of the beginning of LR implementation. Patient sampling in our study contained information from all psychiatric hospitalizations in Israel (13,582 admissions in 2000, of which 3124 were involuntary, and 12,894 admissions in 2010, of which 3434 were involuntary) and was not limited to a specific hospital, as it had been in the earlier two studies. Furthermore, the absence of data presentation and statistical analysis in the first study [19] precludes a meaningful comparison between its findings and ours. The second study [20] found that the duration until readmission was shorter among LR patients if they were brought before a DPB during the first two weeks of admission. However, only two patients were released by appeals in 2003–2004 compared with eleven LR patients in 2005. Time until readmission in that study was also much different from ours: it was an average of 472 days for unrepresented patients and 264 days for represented. Methodological flaws notwithstanding, the paucity of data available on this issue and the contradictory results between those studies and ours warrant additional research.

### Research limitations

The major limitations of our study are that it used data from only two time points, and that it presents data acquired fourteen years ago. Since there is a ten-year gap between the two time points we examined, changes other than the initiation of LR that had occurred during this time period may have affected discharge rates by DPBs, such as the development of novel medications, modifications in admission procedures, amendments in mental health policy reforms, and changes in the number of available hospital beds [8]. In addition, while data from the Israeli National Psychiatric Hospitalization Registry for 2000 and 2010 were complete with regard to the mode of discharge (by TP or a DPB) of involuntarily hospitalized patients, they included only partial data on the overall number of DPB proceedings per year, and none on the number of DPB proceedings per patient, on whether the proceedings were convened to appeal the commitment or to recommend an extension of hospitalization, or on the outcome of each proceeding. This missing information further limited the scope of our findings. Fortunately, these data are now available and should be used for future studies. Finally, while a study design best suited for evaluating the effect of LR upon readmission and DPB discharge rates would be a prospective analysis for comparing random allocation vs. non-allocation of LR, it would be unethical to withhold LR from involuntarily committed psychiatric patients for the sole purpose of research.

### Recommendations for further research

In light of the limitations of the current work, we recommend that the data we extracted from years 2000 and 2010 be compared to LR related changes from 2020. Updated data now available at the Israeli National Psychiatric Hospitalization Registry are more comprehensive and may allow the monitoring of additional variables, such as the number of yearly DPB proceedings, the effect of patient diagnosis on the outcomes of the proceedings, and a comparison between outcomes for proceedings appealing the commitment and those recommending its extension. Such corroboration is warranted to further validate our findings.

## Conclusions

There has been a significant increase in the number of DPB proceedings and discharges of psychiatric patients involuntarily hospitalized by DPBs since the introduction of state-funded LR in Israel in 2004. However, our findings showed no changes in thirty-day and six-month readmission rates. Thus, we conclude that state-funded LR does not compromise beneficence and non-malfeasance of patient ca. Our results underscore the feasibility of granting even the most severely mentally ill patients the right to due process. We hope that our findings will serve to relieve concerns about state-funded LR procedures in the setting of involuntary psychiatric hospitalization.

## Supplementary Information

Additional file 1.

Additional file 2.

## Data Availability

The data in this study are available from the corresponding author upon reasonable request. Anonymous already collected database from the Israeli National Psychiatric Hospitalization Registry of the Ministry of Health.

## Abbreviations

LR: Legal representation
MHA: Mental Health Act 1991
DPB: District Psychiatric Board
RD: Revolving Door
TP: Treating Psychiatrist
AMA: Against medical advice
OR: Odds ratio
