# Supplementary material for: The effect of legal representation on clinical measures in involuntarily admitted psychiatric patients: a retrospective study
Source: Isr J Health Policy Res. 2024 Oct 3;13:58. doi: 10.1186/s13584-024-00633-9 (PMC11448244; doi:10.1186/s13584-024-00633-9)
Supplement: Supplementary file 1 — Additional file 1. [file 13584_2024_633_MOESM1_ESM.docx]

**Supplementary Figure 1.** Distribution of hospitalization stays for involuntary hospitalizations.

(a) Histogram depicting the fraction of hospitalizations (y-axis) according to the length of stay (weeks, x-axis), grouped by type of discharge (treating psychiatrists [TP] vs. a District Psychiatric Board [DPB]) and by study year (2000 vs. 2010). (b) Same data following log_2_-transformation.

**Supplementary Figure 2.** Distribution of hospitalization stays for voluntary admissions and its prediction by year. (a) Histogram depicting the fraction of hospitalizations (y-axis) according to the length of stay (weeks, x-axis), grouped by year (2000 vs. 2010). (b) Same data following log_2_-transformation. (c) Bar graph depicting the effects of year (2000 vs. 2010) on mean (±95% CI) duration of hospitalization (log_2_-weeks). The significance value is based upon a non-paired Students’ t-test.
