# Supplementary figures and images for: The effect of legal representation on clinical measures in involuntarily admitted psychiatric patients: a retrospective study
Source: Isr J Health Policy Res. 2024 Oct 3;13:58. doi: 10.1186/s13584-024-00633-9 (PMC11448244; doi:10.1186/s13584-024-00633-9)

## Slide 1
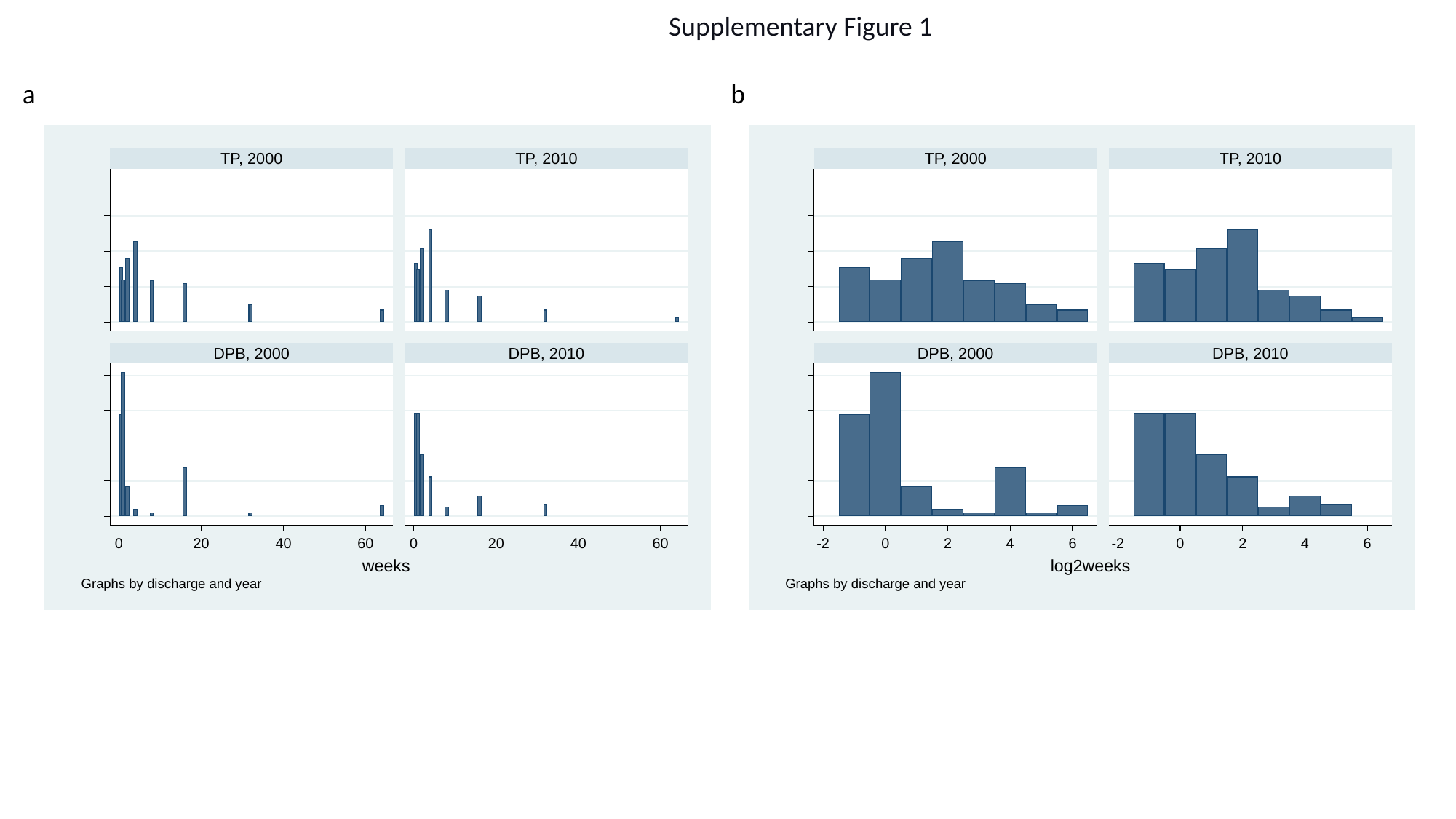

Supplementary Figure 1
a
b
b

## Slide 2
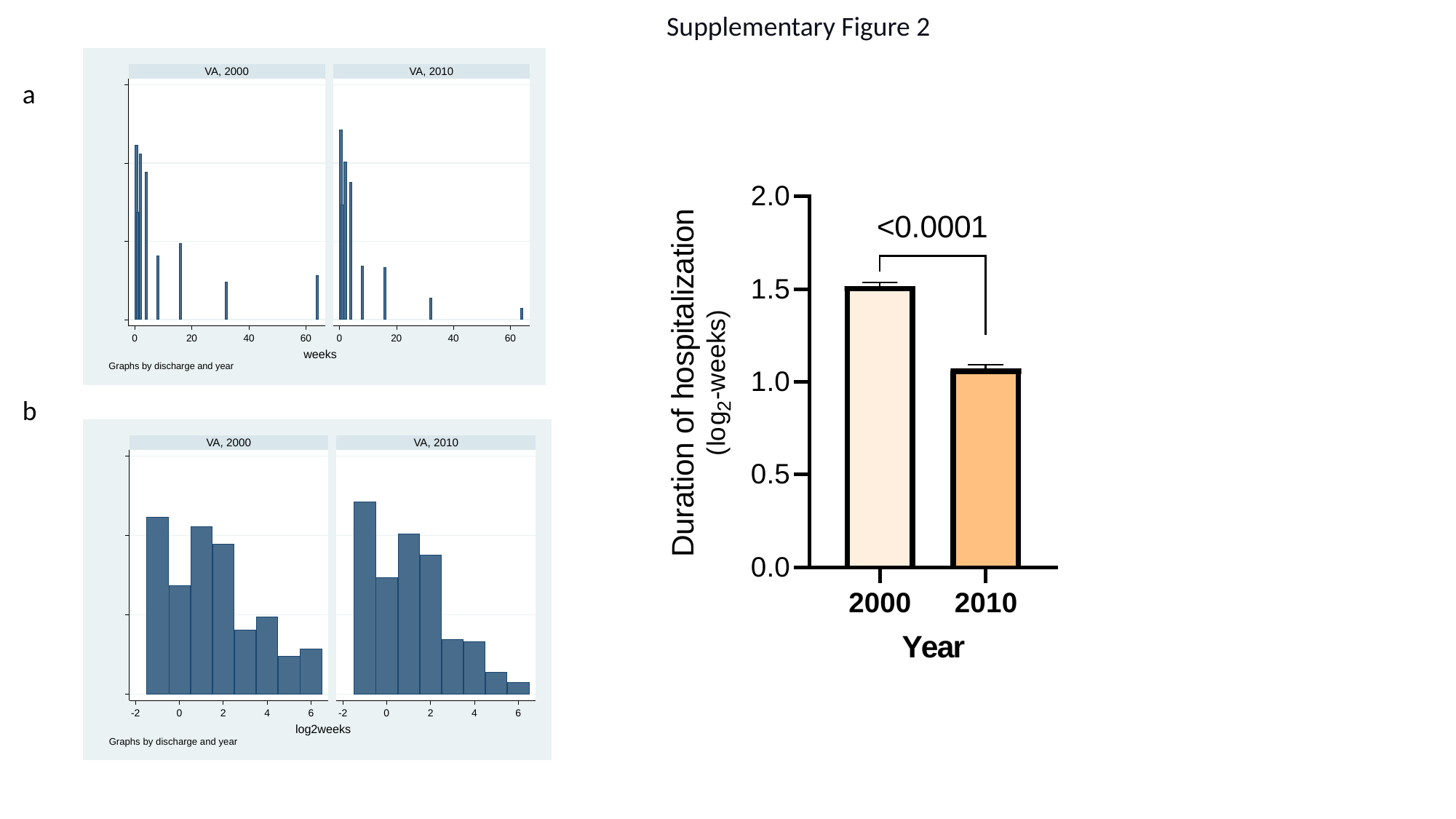

Supplementary Figure 2
a
b

Supplement: Supplementary file 2 — Additional file 2. [file 13584_2024_633_MOESM2_ESM.pptx]
